# That dog won’t fit: body size awareness in dogs

**DOI:** 10.1007/s10071-019-01337-3

**Published:** 2019-12-12

**Authors:** R. Lenkei, T. Faragó, D. Kovács, B. Zsilák, P. Pongrácz

**Affiliations:** Department of Ethology, Institute of Biology, Eötvös Loránd University, Pázmány Péter sétány 1/c, Budapest, 1117 Hungary

**Keywords:** Self-representation, Body awareness, Body size awareness, Dog, Modular structure

## Abstract

With very few exceptions, no coherent model of representing the self exists for nonhuman species. According to our hypothesis, understanding of the Self as an object’ can also be found in a wide range of animals including the dog, a fast-moving terrestrial predator/scavenger, with highly developed senses and complex cognitive capacity. We tested companion dogs in three experiments in which they faced three different variations of the same physical challenge: passing through an opening in a wall. We predicted that if dogs are capable of representing their own body size, they will react differently when faced with adequate or too small openings. We found that dogs started to move towards and approached the too small openings with significantly longer latencies than the suitable ones; and upon reaching it, they did not try to get through the too small openings. In another experiment, the medium-size (still large enough) opening was approached with latencies that fell between the latencies measured in the cases of the very large or the too small openings. Having discussed the potential underlying mechanisms, we concluded that our results convincingly assume that dogs can represent their own body size in novel contexts.

**Electronic supplementary material:**

The online version of this article (10.1007/s10071-019-01337-3) contains supplementary material, which is available to authorized users.

## Introduction

When considering whether non-human animals can show signs of self-representation, while admitting that humans possess the most complex form of self-consciousness that emerges with the development of linguistic abilities (Bekoff 2002), researchers usually try to find evidence for capacities in various species that would show a close match with the highest non-verbal manifestations of self-awareness in humans. A non-exclusive list of these includes the theory of mind (e.g.: in chimpanzees: Premack and Woodruff 1978; in dolphins: Tomonaga and Uwano 2010, empathy (e.g.: in elephants: Byrne et al. 2008; in chimpanzees: Anderson et al. 2004) or mental time-travel (e.g.: in scrub-jays: Clayton et al. 2003; in dogs: Fugazza et al. 2016).

The traditional comparative approach (in chimpanzees: Gallup 1970; in bottlenose dolphins: Reiss and Marino 2001; in Asian elephants: Plotnik et al. 2006; in Eurasian magpies: Prior et al. 2008) is mostly based on the well-known, although often contested, mirror mark test paradigm, introduced by Gallup (1970), which leads almost inevitably to an arbitrarily restricted view of non-human self-representation capacity (e.g.: in gorillas: Ledbetter and Basen 1982; in giant pandas: Ma et al. 2015; in African gray parrots: Pepperberg et al. 1995). Besides some argued weaknesses of the paradigm itself (e.g.: Epstein et al. 1981; Heyes 1995; Suddendorf and Butler 2014), to face and observe themselves in a mirror might simply be an inappropriate task for many animals, because of their lack of motivation to examine their own physical appearance or to remove a mark, or because of failure to understand the mirror’s properties (Hauser et al. 2001). The inherent problem of the application of the mirror test to nonhuman species is the possible lack of ecological need for visual self-recognition and complex cognitive self-awareness by these animals (similarly, it is usually also not tested meticulously whether these species use visual cues to recognize their conspecifics). This may lead scientists to consider self-representation in species that did not pass the test, as either non-existing, or incomparably weaker than it is in humans (De Veer and Van den Bos 1999). However, being a product of evolutionary adaptation processes, representing the self must show qualitatively and quantitatively different cognitive manifestations that have evolved to fulfill different ecological needs which in turn would make it likely that its presence can be discovered in a multitude of species (Bekoff and Sherman 2004).

Besides the abundance of theoretical works (e.g.: Edelman and Seth 2009; Morin 2006; Povinelli and Cant 1995; Rochat 2003) the attempt to develop an empirical, bottom-up framework to experimentally test self-representation in various species is mostly lacking. Here we propose a parsimonious approach based on a theory that the self is an abstract multimodal concept (Kaplan et al. 2008). Our approach follows the notion that the ability of self-representation is an array of interconnected cognitive skills, in which the presence of each of these ‘building blocks’ may vary according to the species. Among these components one could mention the awareness of one’s own actions in the past, understanding the relationship between one’s body and the environment, or self-recognition based on different sensory modalities. These components may have evolved differently in each species due to their unique environment and life history (Bekoff and Sherman 2004). This modular approach allows us to test operationally for the presence of different cognitive traits that may belong to the representation of the self in different species. In the future, it will eventually allow comparative conclusions on the evolution of more complex and structured abilities to represent the self in humans, to be drawn.

The modular framework of self-representation also indicates that the most complex forms of this capacity should emerge in those cases of long-lived, highly social animals where the individuals engage in repeated interactions with each other (Bekoff and Sherman 2004). We have experimental evidence supporting this assumption, as positive examples were found in the case of primates (Anderson and Gallup 1999); dolphins (see for a review: Herman 2012) and also in elephants (Dale and Plotnik 2017; Plotnik et al. 2006). However, we do not know about a comprehensive animal model so far, where each of the possible modules connected to self-representation would be investigated from an evolutionary and ecologically appropriate point of view. We propose the dog as an optimal model to systematically investigate the various cognitive traits connected to self-representation because of its unique evolutionary history and social environment. The dog occupies a special niche living in the highly complex human-environment forming heterospecific social groups with humans (Miklósi and Topál 2013). Furthermore, there is ample evidence which shows that dogs have complex socio-cognitive skills that enable them to partake in an array of inter-specific interactions with humans. More importantly, these capacities of dogs involve such cognitive traits that are considered to be important in the case of differing aspects of representing the self, or representing others’ goals/intentions or emotions. The latter also can be important when distinguishing between the self and others. Among others, it was shown that dogs are capable of social learning (e.g. Pongrácz et al. 2001), including various instances of imitation (Topál et al. 2006) where in some specific cases we have convincing evidence for imitating novel actions through episodic memory as well (Fugazza et al. 2016). Dogs not only have a given identity, but they are also able to recognize their own names, even amidst distracting verbal background noise (Mallikarjun et al. 2019). Dogs are sensitive to the attentional states of humans (Gácsi et al. 2004) and they also readily follow various visual referential cues (e.g.: Miklósi et al. 1998). They are capable of knowledge-attribution to humans (Virányi et al. 2006), and they are sensitive to various manifestations of human emotions (e.g. contagious yawning: Romero et al. 2013; emotional vocalizations: Huber et al. 2017; visual expressions: Turcsán et al. 2015). Thus, it is reasonable to assume that dogs may possess a complex enough mental representational system and also an ecologically valid need for at least some form of representing the self. In the case of dogs, there were sporadic efforts where some components of self-representation were tested, such as the presence of episodic-like memory (Fugazza et al. 2016). It was another approach, when they investigated the ability of the discrimination of own odor from others’ (Bekoff 2001; Gatti 2016; Horowitz 2017). These studies are based on the concept that dogs’ primary sense is not vision what is tested in the mirror mark test but olfaction, so the olfactory cues would represent more relevant stimuli to this species. During the so-called “olfactory mirror test”, dogs were presented with canisters containing urine samples either from themselves, other familiar or unfamiliar dogs’, or their own urine sample with added odor. They found that dogs spent more time investigating other dogs’ odor than their own, and they also sniffed longer their own modified sample than the one without the added odor (Horowitz 2017). Although this approach is promising and has ecological validity, it is hard to exclude that dogs would spend more time investigating new or more complex, i.e. modified odors because of their novelty-effect. The other weakness of the test is that it does not imply any interaction with the dogs’ own body. If dogs could identify the odors they should have smelled themselves after smelling the modified samples analogously to the subjects who touch the mark on their body in the mirror test (Gallup and Anderson 2018). Although studies about self-recognition by using different modalities can be important, from the evolutionary point of view self-representation could more likely manifest itself during locomotion (Povinelli and Cant 1995; Moore et al. 2007). Thus, in this paper we investigated the ability of dogs to represent their own body size, as active locomotion in the physical environment poses a widespread and fundamental challenge for numerous animal species; therefore, it offers a good starting point for testing the modular structure of self-representation (Bekoff 2002).

For multicellular, large-bodied organisms with the capacity of active locomotion in their environment, it is vital to be able to overcome or avoid physical obstacles. Simple obstacle-avoidance can be achieved through various senses (e.g. mechanosensation (in cockroaches: Baba et al. 2010); or echolocation: Busnel 2013). For example, in various mammals the vibrissae take a particularly important role in locomotor activities. It was extensively studied in rats (Gustafson and Felbain-Keramidas 1977) and it has been found that the rats were able to assess the width of different apertures with their large vibrissae without active whisker movements (Krupa et al. 2001) and they also use it to determine distances in the dark (Jenkinson and Glickstein 2000). There is no doubt that the whiskers have an important role in case of dogs as well, although unfortunately there is no behavioral data about whether dogs rely on their whiskers while navigating in the physical environment (McGill 1980).

With a necessarily complex neural background, the theoretical possibility for self/environment discrimination may emerge as well (Neisser 1995 ‘ecological self’). The next evolutionary step towards a more developed representation of the self in an individual is called ‘objective self’ (Povinelli and Cant 1995), where ‘body awareness’, which is “the ability to hold information about one’s own body in mind as an explicit object of attention in relation to other objects in the world” (Brownell et al. 2007), serves as the most fundamental building block. A good example for storing information about one’s own body, and one of the most fundamental bases of self-representation, is that human children are able to distinguish a live film of their own and another child’s moving legs, by 5 months of age, significantly earlier than linguistic skills would emerge (Bahrick and Watson 1985).

Body image in adult humans is a multimodal concept, consisting of a perceptual component which includes the unconscious process of proprioceptive and visual information of the body’s spatial position, the awareness of the structure and shape of the body, and finally its visual appearance. The other conscious component provides the capacity of having an attitude towards the physical self, including the thoughts and feelings about one’s own body, which in turn influences the individual’s psychology and behavior (Haggard and Wolpert 2005; Irvine et al. 2019; Stice and Shaw 2002). Human babies are born with the immediate capacity to explore their own bodies (Bahrick and Moss 1996). The earliest emerging ability during their development is the understanding of the relationship between proprioceptive and visual consequences of the motion of their own limbs (Bahrick and Watson 1985). The ability to understand the physical characteristics of their own body develops gradually during the first years of life (e.g. Brownell et al. 2007; Franchak and Adolph 2012; Moore et al. 2007). To study the perceptual component of body-awareness in children, as well as in adults, different versions of the ‘door choice task’ serve as a commonly used paradigm (e.g.: Boyer et al. 2012; Brownell et al. 2007; Irvine et al. 2019; Stefanucci and Geuss 2009). For example, Warren and Whang (1987) determined the critical aperture-to-shoulder width ratio, concluding that in the case of adult humans, the threshold ratio is A/S = 1.3, because if the aperture was narrower than this, people would turn their torso before walking through.

In this paper we present the results of three experiments, in which we systematically tested whether dogs rely on an already existing representation of their own size while negotiating physical challenges. In various settings dogs had to get through larger or smaller openings, where before the arrival to the opening they had the chance to decide whether it was large enough for them to pass through but only if they possess the knowledge about their own size. We also tried to exclude alternative decision-making mechanisms, such as simple preference for the more conveniently sized opening, relying solely on learning about the suitability of particular opening sizes, or on a priori experience with apertures of various size and shape. We predicted that if dogs are aware of their own size they will: hesitate more when an opening is seemingly too small for them and they will be faster when approaching large openings as compared to the ‘just big enough’ ones.

## Materials and methods

### General procedure

**Fig. 1:**
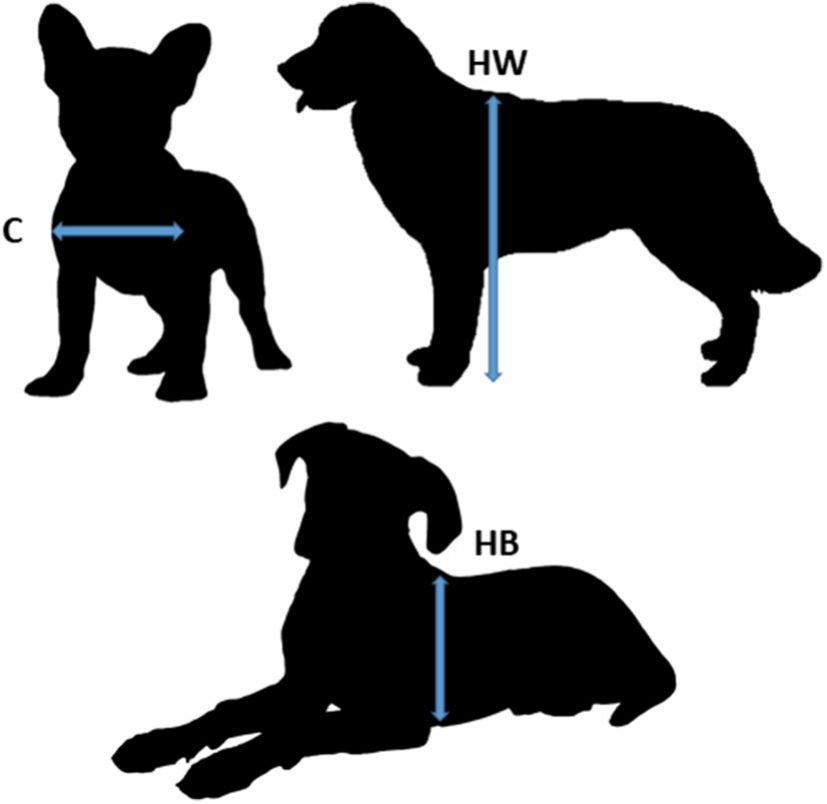
The measurements of the dogs (width of the chest (C); height at the withers (HW), height of the body (HB) in lying position)

The tests were performed indoors, in an experimental room of the Institute. Dogs were taken by their owners to the testing facility, where the owners were also requested to assist during the test with their dog. Before the tests we took the following measurements of the dogs with a measuring tape (in centimetres): width of the chest (C); height at the withers (HW); height of the body (HB) in laying position (Fig. 1). The measuring tape was always held in a taut, straight line while the measurements were taken. These measurements were used for calculating the opening sizes during the tests for each subject individually (see Tables 1, 2 for the opening sizes).

**Table 1:** The list of subjects in Experiment 1

Name	Breed	Sex	Age	Height	Chest width	Small	Medium	Large
Berci	Greyhound	Male	2	66	21	21 × 22	44 × 22	66 × 22
Elza	Greyhound	Female	11	64	21	21 × 21	42 × 21	64 × 21
Bizsu	Labrador Retriever	Female	9.5	60	27	20 × 27	40 × 27	60 × 27
Bogár	Mixed	Female	5	46	18	18 × 18	30 × 18	46 × 18
Jana	Bernese Mountain Dog	Female	1.5	62	25	20 × 25	41 × 25	62 × 25
Cafat	Mudi	Male	1	45	18	15 × 18	30 × 18	45 × 18
Carlo	Greyhound	Male	6.5	70	22	23 × 22	70 × 22	46 × 22
Pufi	Mixed	Male	3	58	21	19 × 21	38 × 21	58 × 21
Csoki	Mixed	Female	2	53	17	17 × 17	35 × 17	53 × 17
Jafar	Mixed	Male	2	57	17	19 × 17	38 × 17	57 × 17
Furfang	Labrador retriever	Male	4.5	58	24	19 × 24	38 × 24	58 × 24
Kiki	Border Collie	Female	3.5	50	24	16 × 24	33 × 24	50 × 24
Lidérc	Malinois	Female	2	61	23	23 × 20	23 × 40	23 × 61
Loki	Labrador Retriever	Male	4.5	70	24	23 × 24	46 × 24	70 × 24
Skala	Saluki	Female	2.5	59	17	19 × 17	39 × 17	59 × 17
Merlin	Labrador retriever	Male	8.5	58	25	18 × 25	38 × 25	58 × 25
Mirza	Springer Spaniel	Female	1	45	18	15 × 18	30 × 18	45 × 18
Luna	Mixed	Female	2	41	14	13 × 15	27 × 15	41 × 15
Máté	Greyhound	Male	2	72	24	24 × 24	48 × 24	72 × 24
Mabon	Border Collie	Male	4	56	21	18 × 21	37 × 21	56 × 21
Panna	Labrador Retriever	Female	9	58	22	19 × 22	38 × 22	58 × 22
Bizsu2	Siberian Husky	Female	3	63	23	21 × 23	42 × 23	63 × 23
Panka	Golden Retriever	Female	8	58	23	19 × 23	38 × 23	58 × 23
Rohan	Border Collie	Female	5	48	20	16 × 20	32 × 20	48 × 20
Velúr	Greyhound	Male	5	81	23	27 × 23	54 × 23	81 × 23
Zara	Greyhound	Female	1.5	66	24	22 × 24	44 × 24	66 × 24
Zloty	Cairn Terrier	Female	2	34	18	11 × 18	22 × 18	34 × 18
Sonny	Greyhound	Male	5	70	25	23 × 25	47 × 25	70 × 25
Carlos	Rhodesian Ridgeback	Male	3	75	32	23 × 32	46 × 32	75 × 32
Füge	Border Collie	Male	1	52	24	17 × 24	34 × 24	52 × 24
Walter	Golden Retriever	Male	6.5	62	24	20 × 24	41 × 24	62 × 24
Floyd	Golden Retriever	Male	3	62	24	20 × 24	41 × 24	62 × 24

The basic information of the subjects in Experiment 1 and the exact opening sizes that they were given during the tests. Age is given in years, all size measurments are given in centimetres

**Table 2:** The list of subjects in Experiments 2 and 3

								Experiment 2						Experiment 3.	
Name	Age	Breed	Sex	Height at the withers	Height of the body	Width of the chest	First Test	1	2	3	4	5	6	1–3	4. test
*Subjects tested in Experiments 2 and 3*															
Álmos	9	Vizsla	Male	72	33	25	Exp 2	72 × 50	59 × 42	46 × 33	33 × 25			60 × 33	33 × 60
Arizona	5	Welsh Corgi	Female	32	22	19	Exp 3	32 × 38	29 × 32	26 × 25	22 × 19			60 × 22	22 × 60
Boglya	4	Vizsla	Male	69	33	23	Exp 2	69 × 46	57 × 39	45 × 31	33 × 23			60 × 33	33 × 60
Diego	4	GSD	Male	65	35	27	Exp 3	65 × 54	52 × 45	43 × 36	35 × 27	17 × 18		60 × 35	35 × 60
Fox	2	Welsh Corgi	Male	36	23	26	Exp 3	36 × 52	32 × 44	27 × 35	23 × 27	19 × 17		60 × 23	23 × 60
Gustav	3	Welsh Corgi	Male	30	21	22	Exp 3	30 × 44	25 × 29					60 × 21	21 × 60
Jenny	3	Irish Setter	Female	66	30	20	Exp 2	66 × 40	54 × 33	42 × 26	30 × 20			60 × 30	30 × 60
Kecmec	3	Dachshund	Female	31	19	20	Exp 3	31 × 40	27 × 34	23 × 27	19 × 20	15 × 14	11 × 7	60 × 19	19 × 60
Lizzy	5.5	GSD	Female	60	36	25	Exp 3	60 × 50	52 × 44	44 × 34	36 × 25	28 × 17		60 × 36	36 × 60
Loki	2	Welsh Corgi	Male	28	24	21	Exp 3	28 × 42	25 × 35					60 × 21	21 × 60
Lola	5	Vizsla	Female	60	26	22	Exp 2	60 × 44	49 × 37	37 × 29	26 × 22			60 × 26	26 × 60
Málna	2	Dachshund	Female	30	17	17	Exp 3	30 × 34	26 × 29	21 × 23	17 × 17	12 × 11		60 × 17	17 × 60
Pepe	6	Dachshund	Male	33	20	20	Exp 2	33 × 40	29 × 34	24 × 27	20 × 20	16 × 13		60 × 20	20 × 60
Rozi	4	Dachshund	Female	20	15	10	Exp 3	20 × 30	18 × 25					60 × 15	15 × 60
Rudi II	2.5	Vizsla	Male	60	28	23	Exp 2	60 × 46	50 × 39	39 × 31	28 × 23	18 × 15		60 × 28	28 × 60
Sumák	4	Greyhound	Male	62	30	24	Exp 2	62 × 48	50 × 40	41 × 32				60 × 30	30 × 60
Szofi	6	Welsh Corgi	Female	29	21	24	Exp 2	29 × 42	26 × 34	24 × 28	21 × 24	18x15		60 × 24	24 × 60
Wolfie	2.5	Belgian Shepherd	Male	65	30	15	Exp 3	65 × 30	53 × 25	42 × 20				60 × 30	30 × 60
Zorro	10	Vizsla	Male	65	30	27	Exp 2	65 × 56	54 × 45	42 × 36	30 × 27	18 × 16		60 × 30	30 × 60
Subjects tested only in Experiment 2															
Anouk	1.5	Standard Poodle	Female	62	23	23	Exp 2	60 × 46	47 × 39	35 × 31	23 × 23	12 × 16			
Bejgli	2.5	Beagle	Male	50	25	18	Exp 2	50 × 36	41 × 30	33 × 24	25 × 18				
Cara	5	Greyhound	Female	69	31	20	Exp 3	60 × 40	50 × 34	40 × 27	32 × 10			Excluded	
Cimpa	1.5	Mixed	Male	47	25	22	Exp 2	47 × 44	40 × 37	32 × 29	25 × 22	18 × 15	11 × 7		
Csicsi	8	Mudi	Female	44	19	17	Exp 2	44 × 34	38 × 30	32 × 26	26 × 22	20 × 18			
Dolores	4	Mixed	Female	50	25	17	Exp 3	50 × 34	42 × 29	34 × 23	25 × 17	18 × 11			
Igor	2	Greyhound	Male	70	35	25	Exp 2	70 × 50	58 × 42	47 × 33	35 × 25				
Jóság	11	Mudi	Female	49	20	16	Exp 2	49 × 32	42 × 28	35 × 24	28 × 20	21 × 16			
Kiwi	2.5	Greyhound	Female	57	25	19	Exp 2	57 × 38	46 × 32	36 × 25	25 × 19				
Lara	4	Border Collie	Female	48	26	16	Exp 2	48 × 32	40 × 26	31 × 21	22 × 16				
Mazsola	5	Dachshund	Female	26	22	16	Exp 3	26 × 32	25 × 27	24 × 21	21 × 16	18 × 11	16 × 6	Excluded	
Millie	10	West Highland White Terrier	Female	30	19	16	Exp 2	30 × 32	27 × 26	23 × 21	19 × 16				
Negev	1.5	Dachshund	Male	28	20	20	Exp 2	28 × 40	25 × 36	23 × 29	20 × 20				
Nuance	1.5	Dachshund	Female	22	17	16	Exp 2	22 × 44	20 × 37	18 × 29	16 × 22				
Patrik	3	Dachshund	Male	27	20	15	Exp 3	27 × 30	25 × 25	22 × 20	20 × 15			Excluded	
Puszedli	7	Mixed Breed	Female	39	21	19	Exp 2	39 × 42	33 × 35	27 × 28	21 × 21	15 × 13			
Remi	10	Mudi	Female	44	21	17	Exp 2	44 × 32	38 × 28	32 × 24	26 × 20	20 × 16	14 × 12		
Süti	3	Beagle	Female	39	21	19	Exp 2	39 × 38	33 × 32	27 × 25	21 × 19	15 × 13			
Süti2	4	French Bulldog	Female	29	22	23	Exp 3	29 × 46	26 × 39	24 × 31	22 × 23	17 × 15			
Tira	7	Mudi	Female	51	22	18	Exp 2	51 × 36	44 × 32	37 × 28	30 × 24	23 × 20			
Viktória	2	Mixed	Female	62	32	27	Exp 2	62 × 54	52 × 45	42 × 36	32 × 27				
Vilmos	2.5	Vizsla	Male	65	31	23	Exp 3	65 × 45	54x39	42 × 31	30 × 23	19 × 16		Excluded	
Vince	5	Standard Poodle	Male	63	27	23	Exp 2	63 × 51	51 × 39	39 × 31	27 × 23	15 × 16			
*Subjects tested only in Experiment 3*															
Brutus	1	GSD	Male	68	32	25	Exp 2		Excluded					60 × 32	32 × 60
Csanád	3	Vizsla	Male	67	29	24	Exp 3							60 × 29	24 × 60
Csoki	6	Dachshund	Male	26	20	19	Exp 3							60 × 20	20 × 60
Fifi	9	Dachshund	Female	23	19	16	Exp 3							60 × 19	19 × 60
Frida	3.5	Basset Hound	Female	35	27	27	Exp 3		Excluded					60 × 27	27 × 60
Lizzie	5	Dachshund	Female	31	20	20	Exp 3							60 × 20	20 × 60
Mici	9	Vizsla	Female	51	26	21	Exp 3		Excluded					60 × 26	26 × 60
Pullo	6	Vizsla	Male	66	35	24	Exp 3							60 × 35	35 × 60
Rebeka	3.5	Vizsla	Female	63	34	28	Exp 3		Excluded					60 × 34	34 × 60
Rudi	4	Vizsla	Male	66	28	26	Exp 2		Excluded					60 × 28	28 × 60
Simon	4	Dachshund	Male	25	18	16	Exp 3							60 × 18	18 × 60
Sissy	5.5	Dachshund	Female	24	15	15	Exp 3							60 × 15	15 × 60
Wassabi	3	GSD	Female	69	34	24	Exp 3							60 × 34	34 × 60
Wendy	4	Welsh Corgi	Female	30	23	22	Exp 3							60 × 23	23 × 60
Wini	1.5	Welsh Corgi	Male	31	24	18	Exp 2		Excluded					60 × 24	24 × 60
Woody	1.5	Welsh Corgi	Male	31	21	25	Exp 3							60 × 1	21 × 60

The basic information of the subjects of Experiments 2 and 3 and the exact opening sizes they were facing during the tests. In Experiment 2 the maximum amount of trials varies according to the number of steps we had to gradually downsize the opening until the dog did not go through it. Age is given in years, all the measurements are given in centimetres. *GSD* German Shepherd Dog

**Fig. 2:**
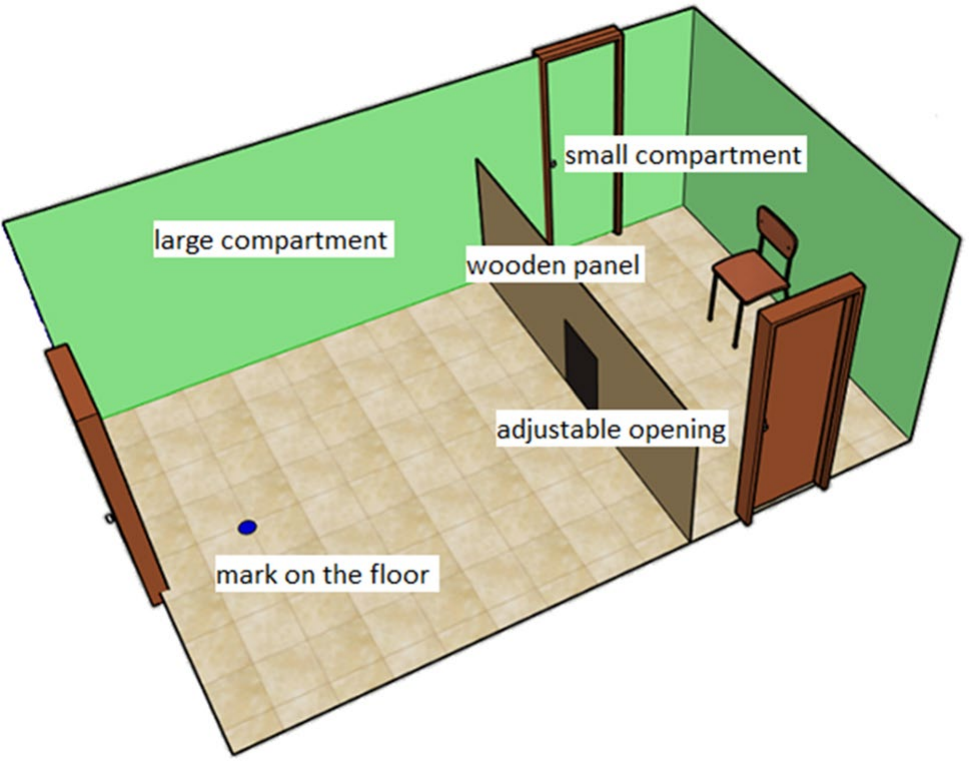
The schematic outlay of the experimental room

The testing room (5.2 m × 3 m) was equipped with three cameras for continuous recording. A wooden panel (height: 125 cm) was set up across the room (see Fig. 2 for the layout). In the middle of the wooden panel we created an adjustable opening that was possible to be set to various widths and heights with the help of two plywood sheets sliding between horizontal and vertical rails. Fast clamps were used to fix the plywood sheets in the required position during the test trials. In the smaller compartment, we positioned a chair in front of the opening, 1.2 m away from it.

### Data analysis

The behavior coding of the video footages was performed in Solomon Coder (beta 17.03.22 copyright by András Péter).

We analyzed the latency of leaving the start mark (in Experiments 2 and 3), the latency of arriving to the opening, and the passing-through attempts as a binary variable. An independent observer coded 10-10 video footages from each experiment. The reliability of coding was analyzed by Spearman’s Rho-correlation. Based on the analysis our coding procedure was reliable (Experiment 1. arrive: rs = 0.944; *p* < 0.001; Experiment 2 leaving the start mark: rs = 0.851, *p* < 0.001; arrive: rs = 0.964, *p* < 0.001; Experiment 3: leaving the start mark: rs = 0.638, p < 0.001; arrive: rs = 0.859, *p* < 0.001).

### Statistical analysis

The analysis was performed in R (R Core Team 2016) by using coxme package. In the case of latencies, we used Mixed Effects Cox Regression (coxme function). Number of crossing attempts was analyzed with generalized linear mixed model (GzLMM), with using Poisson distribution and log link (glm function). For the pair-wise comparisons, we ran Tukey-post hoc tests (emmeans package). We reported the results of the final models. We compared the proportion of dogs that attempted to get through the opening between trials with Chi square test.

### Experiment 1: dogs’ response to an ambiguous size opening after they gained experience with large and too small openings

#### Subjects

We tested *N* = 39 dogs of various breeds and sizes, from which seven were excluded due to the interruption of the test, errors in the experimental procedure or problems with the video recordings. Thus our final subject number was *N* = 32 adult companion dogs (mean age: 3.7 ± 2.6 years, sex ratio: *N* = 16 males and *N* = 16 females). For the main parameters and assignment of the subjects, see Table 1. These subjects participated only in Experiment 1.

#### Procedure

In a warm-up trial, which was identical with the subsequent test trials the opening was set to its maximum size (60 cm wide × 95 cm high) to allow the dog and the owner to become familiar with the nature of the task. In the first 12 trials we provided the subjects with a large enough opening (width of the chest × height at the withers) and also too small openings (width of the chest × $\frac{1}{3}$ × height at the withers) in a semi-randomized order (the first two trials were always different and there were no more than two consecutive trials with the same size opening). For the very last trial, the size of the opening was set halfway between the large and too small opening size (width of the chest × $\frac{2}{3}$ × height at the withers). This medium-sized opening was still large enough for the dog to go through. From the total 13 trials, the first ten served to habituate the dogs to which openings are suitable or not. The last three trials were used for testing the dogs’ reaction to the novel, medium-sized opening.

Before the test, the dog was allowed to explore the area (‘larger compartment’); meanwhile the opening was totally shut by the plywood sheets. Then the owner led the dog back out of the room. Throughout the whole test Experimenter 1 (E1) and Experimenter 2 (E2) stayed in the smaller compartment for adjusting the opening and to call the dog. While sitting on the chair, the legs and feet of E1 were visible from the larger compartment through the opening. E2 stood beside the panel during the entire test because of safety reasons (e.g. if a dog would vigorously try to get through a too narrow opening, in theory it could ruin the whole panel). After 60 s (thus leaving enough time for setting the opening) the owner returned with the dog to the larger compartment. The owner positioned the dog on the start point (3.4 m from the panel). When E2 saw that the dog was ready to be released, she gave a sign to E1, who loudly called the dog (by saying once the dog’s name and the word “come” in Hungarian). Because of the height of the wooden panel, E1 could not see either the dog or the owner while sitting on the chair. In the moment E1 called the dog, the owner released it. The dog was allowed 10 s to try and get through the opening, while the owner was asked to stand passively and in silence. If the dog got through the opening E1 praised it and gave a food reward (a small piece of sausage). If the dog did not get through the opening in 10 s, the trial was over and the owner led the dog out of the room again for 60 s. Each dog had to complete 13 consecutive trials.

### Results of experiment 1

**Fig. 3:**
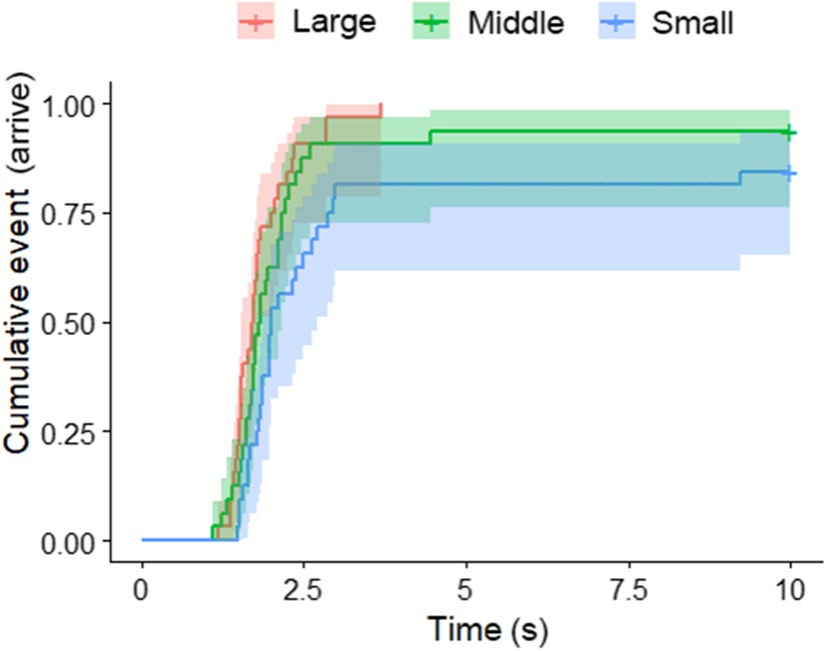
The latency of arriving to the opening in the test trials in Experiment 1. The lines represent the proportion of subjects that had already arrived to the opening at a given point of time elapsed since the start of the trial. Areas shaded with half-tones represent the confidence intervals

We found that during the habituation phase, the dogs arrived significantly sooner to the large opening than to the small ($\chi^{ 2}_{ 1}$ = 192.03; *p* < 0.001). Then during the test phase (when dogs were facing one more time the too small opening, the large enough and additionally the mid-size opening), the latency of arriving to the mid-size opening fell between the two extremities [main effect: ($\chi^{ 2}_{ 2}$ = 39.85; *p* < 0.001); post hoc test: Large → Middle: exp(*β*) = 1.99 (1.43; 2.55); *z* = 2.441, *p* = 0.039; Large → Small: exp(*β*) = 4.92 (3.39; 6.45); *z* = 5.130, *p* < 0.001; Middle → Small: exp(*β*) = 2.48 (1.70; 3.25); *z* = 2.909, *p* = 0.0101 (Fig. 3)]. No effect was shown whether the test phase started with the too small or the large enough opening ($\chi^{ 2}_{ 1}$ = 0.584; *p* = 0.444).

### Experiments 2 and 3

In Experiment 2 dogs of all sizes and builds were included; however, in Experiment 3, the subjects had to be either from an achondroplastic (extreme short legged) breed; or have long legs. Because these tests were rather short, the owners were invited to participate in both. The order of the experiments was randomized across the subjects. Participation in the second test was decided upon the dogs’ tolerance towards separation from the owner between the trials; whether the dog showed any signs of overexcitement or stress during the test. Thus, some subjects were tested only once (see Table 2). In the case of those subjects that participated in both, the two experiments were conducted immediately after each other. As we found no order-effect (see Table 3), we included both tests from the same dogs to the later analysis. All in all, we tested *N* = 65 dogs, but in the case of seven subjects we excluded both tests, while in the case of ten subjects, we excluded only one of the tests from the analysis due to the interruption of the test, errors in the experimental procedure or problems with the video recordings.

**Table 3:** Statistical results of checking the order effect in Experiments 2 and 3

	Chisquare	*df*	*P*
Experiment 2			
Latency to go (L1, L2, L3)	2e–04	1	0.9878
Latency to go (first, L2, L3)	0.0234	1	0.8785
Latency to arrive (L1, L2, L3)	0.8619	1	0.3532
Experiment 3			
Latency to go (all four trials)	0.9577	1	0.3278
Latency to go (last two trials)	0.9577	1	0.3278
Latency to arrive (all four trials)	0.111	1	0.739
Latency to arrive (last two trials)	0.4754	1	0.4905

The results of the models performed to exclude the possible order effect in case of Experiments 2 and 3

In Experiments 2 and 3, the owner sat on the chair in the small compartment instead of the experimenter. As in these experiments the size of the opening was always large enough for the dogs to get through (with one exception in Experiment 2, see later), the motivation level caused by the owner’s presence on the other side of the wooden panel was strong enough for the dogs, even without providing food reward. At the beginning of each trial E2 stood in the smaller compartment while the Owner sat on the chair. E1 in the larger compartment led the dog to the start point, took off the leash and loosened the collar of the dog (loosening of the collar was necessary, because we tested the dogs without collar for safety reasons, and in this way it was possible to release the dog at once when it was called). When E2 saw that the dog was ready to be released, she gave a sign to the owner, who loudly called the dog (by saying once the dog’s name and the word “come” in Hungarian). As soon as the owner called the dog, E1 released it. The dog had maximum 15 s to get through the opening, while the owner had to sit passively and in silence on the other side of the panel. If the dog got through the opening the owner was instructed to praise the dog mildly. No food or toy rewards were allowed. If the dog did not get through the opening in 15 s, the trial was over and E1 allowed the dog get to the owner by fully opening the hole in the panel. Then E1 came back for the dog, took it on leash and led the dog out from the smaller compartment again for the next trial. We left 90 s between the trials, while the Owner and E2 adjusted the new door size.

### Experiment 2: dogs’ response to a gradual size-reduction of opening sizes

#### Subjects

Adult companion dogs (*N* = 42; mean age: 4.4 ± 2.6) of various size, build, and breed (sex ratio: *N* = 18 males and *N* = 24 females).

#### Procedure

In the first trial the height of the opening was equal with HW while the width of the opening was set to 2 °C. During the next trials the opening was gradually downsized based on the following formulas:

$${\text{Vertical}}:\frac{{{\text{HW}} - {\text{HB}}}}{4}$$

$${\text{Horizontal}}:\frac{C}{4}.$$

Trial by trial, the opening was set smaller and smaller till it reached the size where the dogs did not go through (L2). Then in the next (and last, L3) trial the opening was set back to the previous (one step larger) size (i.e. the last opening size where the dog went through before, L1).

### Experiment 3: dogs’ response to differently shaped openings of same size

#### Subjects

Adult companion dogs (*N* = 35, mean age: 4.3 ± 2.2) of long-legged breeds (*N* = 17; sex ratio: *N* = 11 males and *N *= 6 females) and short-legged breeds (*N* = 18; sex ratio: *N* = 8 males and *N* = 10 females).

#### Procedure

1. Habituation phase.
  During the first three trials the opening was 60 cm tall for each subject while the width of the opening was equal with the HB of the given dog.
2. Test phase.
  In the fourth (last) trial we rotated the opening by 90 degrees; thus the width became 60 cm and the height of the opening was equal to the HB of the given dog.

### Results of experiment 2

**Fig. 4:**
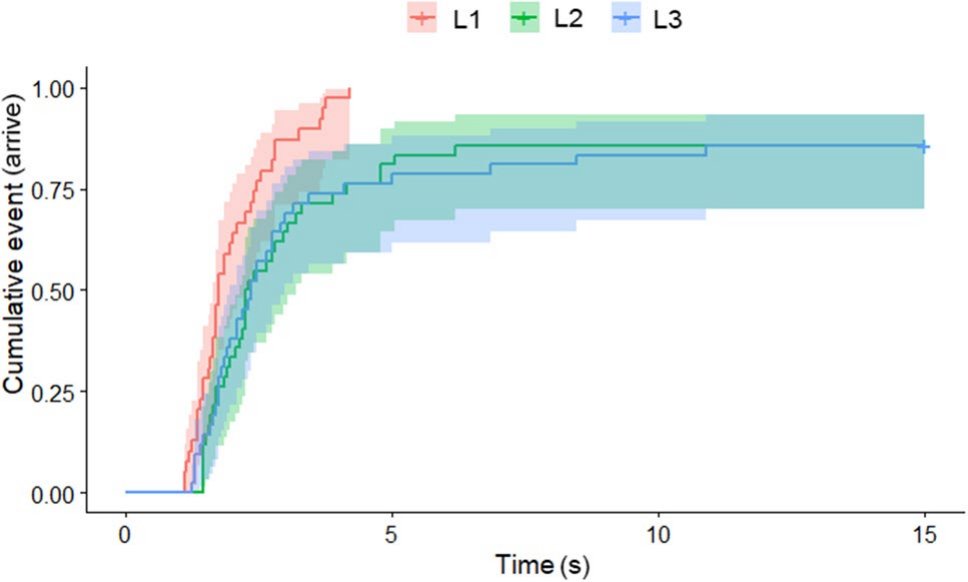
The latency of arriving to the opening in the last three trials in Experiment 2. Lines represent the proportion of dogs that had already arrived to the opening at a given point of time elapsed since the start of the trial. Areas shaded with half-tones represent the confidence intervals

In the case of the latency of leaving the start point, there was no significant difference among the last three trials ($\chi_{2}^{2}$ = 2.652; *p* = 0.265); however, we found a significant trial effect between the first and the last two trials ($\chi_{2}^{2}$ = 7.682; *p* = 0.021). According to the post hoc pairwise comparison, in the first trial dogs started to move sooner than in the last two trials (trial 1 → L2: exp(*β*) = 1.91 [1.44; 2.38]; *z* = 2.61, *p* = 0.024; trial 1 → L3: exp(*β*) = 1.76 [1.34; 2.18]; *z* = 2.33, *p* = 0.051). We found a significant effect when comparing the latencies of arriving to the opening in the last three trials ($\chi_{2}^{2}$ = 31.3; *p* < 0.001). In L1, dogs arrived sooner to the opening, than in L2 and L3 [L1 → L2: exp(*β*) = 4.50 (3.23; 5.77); *z* = 5.33, *p* < 0.001; L1 → L3: exp(*β*) = 4.00 (2.86; 5.13); *z* = 4.884, *p* < 0.001) (Fig. 4). In the case of the attempts to go through, in the L1 trial all the dogs went through, and we found significant difference between L2 and L3. Nineteen subjects did not try to go through in L2 while only six did not try in L3 ($\chi_{2}^{2}$ = 9.624; *p* = 0.001). Fifteen dogs did not try to go through in L2 alone; four dogs did not try to get through both in L2 and L3, and there were only two dogs that did not try to go through only in L3.

### Results of experiment 3

**Fig. 5:**
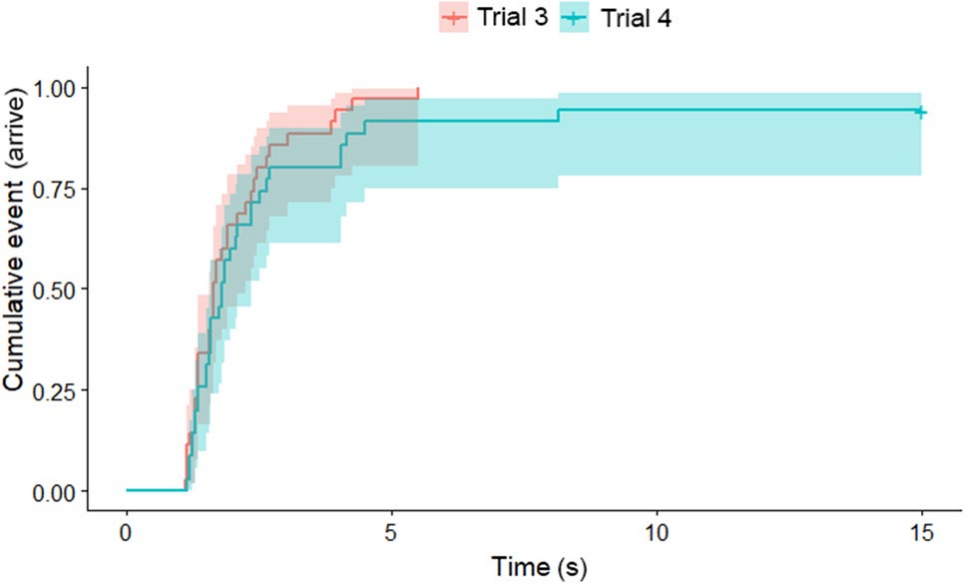
The latency of arrival to the opening in the last two trials in Experiment 3. Lines represent the proportion of dogs that had already arrived to the opening at a given point of time elapsed since the start of the trial. Areas shaded with half-tones represent the confidence intervals

In the case of the latencies of starting to move, we found an interaction between the dog’s height and trial when we compared all four trials ($\chi_{2}^{2}$ = 9.742; *p* = 0.02). Short-legged dogs arrived later in the first habituation trial than in the second [trial 1 → 2: exp(*β*) = 0.26 (0.16; 0.37); *z* = − 3.351, *p* = 0.018]. We did not find significant difference between the last habituation trial and the test trial ($\chi_{1}^{2}$ = 2.762; *p* = 0.096). In the case of arriving to the opening, we found a main effect of trial ($\chi_{3}^{2}$ = 91.148; *p* < 0.001) and height ($\chi_{3}^{2}$ = 9.742; *p* = 0.02) when we compared all four trials. According to the post hoc test, dogs arrived to the opening significantly sooner in the second trial than in the fourth, ‘horizontal opening’ trial [trial 2 → 4: exp(*β*) = 2.28 (1.66; 2.91); *z* = 3.011, *p* = 0.01), and long-legged dogs arrived sooner [short-legged → long-legged: exp(*β*) = 0.31 (0.12; 0.49); *z *= − 1.971, *p* = 0.049]. When we compared only the last two trials ($\chi_{1}^{2}$ = 4.100; *p* < 0.04 (Fig. 5)), we found both a significant trial and height effect. Dogs arrived later in trial 4 (*z* = − 2.25, *p* = 0.025), and short-legged dogs had longer latencies than the long-legged ones (*z* = 2.02, *p* = 0.04).

## Discussion

In a series of experiments where dogs had to pass through a single opening presented on a wall, we found that the size of the opening affected dogs’ behavior both before and during their approach to the opening, and also whether they attempted to get through it. In Experiment 1, similarly to the cognitive bias paradigm (Pogány et al. 2018), dogs were repeatedly exposed to either a too small or a large opening, then at the end they faced a mid-size opening (still large enough to pass through). We found that dogs approached the too small opening significantly later than the large one, and the latency to approach the mid-size opening fell in between. In Experiment 2 the opening was gradually downsized from a comfortably large to a too small opening at which point the dogs did not go through. We found that dogs started to move towards and reached the large enough openings sooner than the one that eventually was proven to be too small. In the final trial, where the opening was enlarged to the last big enough size, significantly more dogs attempted to pass through than in the previous (too small opening) trial. Finally, in Experiment 3 we found that such anatomical features that mostly affect the body proportions, but not the weight of a dog (i.e. achondroplasia), had no effect on how dogs assess the suitability of an opening to pass through. Namely, when we provided dogs with the same size (large enough) rectangular opening in a vertical or horizontal arrangement, we did not find that short-legged dogs approached the horizontal (hence for them still comfortable) opening sooner than the long-legged dogs did.

At this point we know of only one publication where body size awareness (or any sort of body awareness) was tested in dogs (Maeda and Fujita 2010). In that paper the door choice paradigm was used (simultaneously offering two, differently sized doors, both were large enough for the dogs) and authors found a clear preference for the larger door. Those results, therefore, did not indicate body size awareness in dogs, but a possible preference for the more convenient (larger) opening. In the case of human infants, the development of body awareness as a cognitive capacity is usually tested through such erroneous decisions that indicate that children in a given age cohort have more or less difficulty with the representation of their own body as an ‘obstacle’, or they have no clear representation of their own body size (Moore et al. 2007). Brownell et al. (2007), for example, showed that toddlers between 18 and 26 months show a decreasing frequency of the aforementioned errors when trying to pass through an impossibly narrow opening on a wall; meanwhile, they could use a short (0.3 by 0.3 m) opening at this time. In that article, based on the results from four other tasks with the same children, the authors concluded that body awareness develops step by step during the first years of life. We must note, however, that when conclusions are drawn on the basis of only one behavioral parameter (frequency of errors), the resolution of a study is rather low regarding the difficulties of ruling out the alternative explanations. For example, in case of the study of Brownell et al. (2007), it is not known whether the infants made a choice before or during their approach to the openings, or they simply used a trial-and-error strategy. In our experiments with dogs we used multiple parameters that may provide finer details of decision making. By measuring the latency of starting to move towards, and the latency of arriving to the opening, we tackled the possible differences in the a priori decision making of our subjects. Our results are in line with the results of the cognitive bias paradigm where subjects approach the reinforcing stimulus faster than the not reinforcing one and later when they are facing with the ambiguous stimulus they hesitate and the mean latency of approaching falls in between (Mendl et al. 2009, 2010; Pogány et al. 2018). Consequently, when dogs approached a (too small) opening with longer latency, we can conclude that they found it less likely suitable to pass through, and because of the experimental setup, this decision was most probably made by relating the apparent opening size to the mental representation of their own body size. We should remember that ‘too small’ openings except in the habituation phase of Experiment 1 in these experiments were still reasonably ‘big’, calculated by formulas based on the actual size of each individual subject. Additionally, by comparing the attempts to get through the opening in Experiment 2, this showed that when dogs were facing a slightly larger opening after their trial with the too small opening, they did not hesitate to pass through the large enough door. This fact again underlines that dogs decide about the suitability of the individual opening sizes on a case by case basis, likely by using their own body size representation as a template. We must also add at this point that when we mention a ‘template’ of the body size, it is obviously such a mental construct that develops in dogs through a priori encounters with various obstacles beginning from their early ontogeny. However, just because the creation of this template requires experience, it does not mean that the dog has to re-learn each obstacle (i.e. opening size) again and again; on the contrary, the template about its own size makes these types of decisions fast and easy. It is also worthy to mention that the possible connection between experience and the formation of body awareness (i.e. the mental ‘template’) is still unclear even in the case of human infants (see, e.g. Filippetti et al. 2014; Samuels 1986).

In this study our goal was to find evidence in dogs for one of the fundamental building blocks of so-called objective self, body awareness (Moore et al. 2007), while preferably excluding simpler mechanisms for solving the experimental tasks. By providing only one opening at a time, we excluded the option of simply choosing the larger (more convenient, or safer) door (dogs: Maeda and Fujita 2010; children: Brownell et al. 2007), and we did not base our analysis on the number of attempts or the latency of passing through, as we argue that these are mostly dependent on the motivation level of the individual subjects. Similarly, in Experiment 2, we gradually downsized the opening till the subject itself decided that the particular opening size is too small to go through thus we could eliminate the possible differences in the motivation level of the subjects. One could argue that dogs may approach the too small opening with longer latencies because they lost interest in the task towards the end of the experiment; however, we did not find this type of slowing down in the case of the repeated trials with the large enough openings in Experiment 1.

Of course, it is possible that the subjects could try to force themselves through each opening size, and only where they cannot prevail would they give up the attempt. However, we found that this was not the case in Experiment 2, where significantly less dogs even tried to get through the too small opening; meanwhile most of them attempted (and succeeded in) getting through a somewhat larger one. As Franchak and Adolph (2012) underlined, in case of the original door choice tasks, the so-called ‘error’ (i.e. trying to get through the too small opening) has no real, high cost to the individual; consequently, they are not really motivated to avoid it. In children, they found that when the cost was not just getting entrapped in the too narrow opening, but also possibly falling down behind it, the subjects did not try passing through the too small openings. Although in nature entrapment could result in the death of the animal while it tries to squeeze itself through a too narrow opening, there is also the possibility of turning back without serious injury. The results of Franchak and Adolph (2012) supported ours, as dogs did not even attempt to go through if the opening appeared to be too small for them, although the cost would be very low. Furthermore, the latencies of leaving the start point and arriving at the opening showed that dogs distinguished between suitable and unsuitable opening sizes well ahead of actually trying them.

Another possible mechanism that could help the dogs to find out which opening was large enough or too small for them would be the a priori experience with the doors. On the one hand, we could argue that none of our experimental devices were familiar to the dogs; therefore they could not have any knowledge about the suitability of the individual openings. On the other hand, in Experiment 2, where we used all but one opening size only once—except in the last trial dogs did not even have the opportunity to use their freshly gained experience for any of the particular opening sizes coming from the previous trial. Still, it is possible that they would develop some sort of memory-based preference for the ‘conveniently’ sized openings along the serial exposures to the smaller and smaller openings of the actual test; however, this is unlikely because it would result in a steadily increasing latency of approaching. Instead, what we found was a sharp decline of willingness to approach and use the ‘too small’ door in Experiment 2.

Also, one could argue that instead of comparing the size of an actually seen opening to its own body size, dogs with a mechanism different from body-size awareness could somehow estimate the absolute size of an opening and based on that, they could make a decision before they reached the opening in question. The results of Experiment 3 contradict this explanation. Here, dogs faced four times the same size opening, where only the alignment of the opening changed from vertical to horizontal in the last trial. If dogs would mostly rely on a representation of a particular opening size, they would recognize that the two variants are equally large, and they would approach the horizontal opening with the same speed as the vertical ones. However, we found that dogs arrived to the horizontal opening later than to the vertical ones. Another alternative mechanism could be that instead of the size of the opening’s surface, dogs base their decision on the height and width of the opening, and when we ‘rotated’ the vertical opening to the horizontal alignment, the height of the new opening fell into the less suitable category resulting in a slower approach from the subjects.

Another possible explanation is that dogs might simply learn about particular opening sizes during their everyday interactions with their physical environment; thus in our experiments they could rely on their positive or negative experiences from the past and when they go through a new opening they compare its size with the previously learned sizes. Although learning from the experiences of interactions with the physical environment is plausible during the development of the own body size template, based on our results we argue that in our case not only external cues, i.e. learning about the particular openings during the tests shaped the decision making of the dogs. In Experiment 3, we found that short-legged dogs arrived to the horizontal opening later than the long-legged dogs. If dogs’ responses would mostly depend on previous experiences regarding suitable openings, we would expect just the opposite: short-legged dogs would remain similarly fast regardless of the alignment of the opening (as the horizontal opening was still comfortably high for them); meanwhile long-legged dogs would slow down due to the ungainly alignment of the horizontal opening. As our results showed the opposite, the theory of previous experience-driven decision making is less likely; instead, the later arrival to the opening in both groups can be rather explained with the effect of surprise (i.e. the alignment of the opening had been changed), and also with the possibly slower locomotion of the short legged dogs. Similarly, in Experiment 2 during the last trial when the door size was enlarged again one could expect that dogs should have been as fast as when they were facing that particular size for the first time. In other words, if dogs would rely only on their past experiences regarding the opening sizes, they would pass through the large enough door sooner in the last trial than in the too small one just before. However, we found that there was no difference between the latencies in the case of the last two trials probably because of the negative experience of facing a too small opening in the previous trial. This hesitation may also support the existence of a priori decision making of the dogs before they actually approach an opening.

## Conclusion

Dogs represent a suitable model for studying systematically the modular construction of self-representation from its more basic to its more complex manifestations with a strong emphasis on the ecological and evolutionary aspects. Body size-awareness is considered to be one of the simpler, yet fundamental building blocks of self-representation, which is especially important in the case of large-bodied animals that live in complex environments (Povinelli and Cant 1995). With the help of our experiments we could exclude several possible alternative mechanisms behind the observed behavioral responses of dogs while negotiating the ‘get through an opening’ task; therefore, we argue that dogs are able to represent their body size and they do not necessarily rely only on learning about particular openings or external cues; thus they make an a priori decision about the suitability of a particular opening. In case of dog breeds with extreme anatomical features (such as the dwarfishly short legs) it is still not known whether these dogs develop a mental ‘body size template’, which acknowledges the unusually short stature of these dogs, or the mental template of these dogs resembles rather the size of a dog of similar weight but with legs of normal length. As a possible future direction of experimentation, the ontogenetic aspect of body-awareness would be worthy to investigate in the proposed canine model, where it would be possible to study the gradual development of the knowledge about one’s own body through its relationship with the environment.

## Electronic supplementary material

Below is the link to the electronic supplementary material.

Supplementary material 1 (XLSX 21 kb)

Supplementary material 2 (XLSX 34 kb)

## Data Availability

The datasets generated and analyzed during this study are available as electronic supplementary material.
